# Large-scale synchronized activity in the embryonic brainstem and spinal cord

**DOI:** 10.3389/fncel.2013.00036

**Published:** 2013-04-05

**Authors:** Yoko Momose-Sato, Katsushige Sato

**Affiliations:** ^1^Department of Health and Nutrition, College of Human Environmental Studies, Kanto Gakuin UniversityYokohama, Japan; ^2^Department of Health and Nutrition Sciences, Faculty of Human Health, Komazawa Women's UniversityTokyo, Japan

**Keywords:** spontaneous activity, embryo, brainstem, spinal cord, development, synchronization

## Abstract

In the developing central nervous system, spontaneous activity appears well before the brain responds to external sensory inputs. One of the earliest activities is observed in the hindbrain and spinal cord, which is detected as rhythmic electrical discharges of cranial and spinal motoneurons or oscillations of Ca^2+^- and voltage-related optical signals. Shortly after the initial expression, the spontaneous activity appearing in the hindbrain and spinal cord exhibits a large-scale correlated wave that propagates over a wide region of the central nervous system, maximally extending to the lumbosacral cord and to the forebrain. In this review, we describe several aspects of this synchronized activity by focusing on the basic properties, development, origin, propagation pattern, pharmacological characteristics, and possible mechanisms underlying the generation of the activity. These profiles differ from those of the respiratory and locomotion pattern generators observed in the mature brainstem and spinal cord, suggesting that the wave is primordial activity that appears during a specific period of embryonic development and plays some important roles in the development of the central nervous system.

## Introduction

The central nervous system is an information-processing system that receives external sensory inputs and integrates them with endogenous spontaneous activity to provide appropriate behavioral outputs. During development, embryos show spontaneous movements well before sensory pathways are functionally organized (Movie S1). This behavior, called embryonic motility, was originally described more than a century ago (Preyer, 1885) and has been best characterized in chicken embryos by Hamburger and colleagues (for reviews see Bekoff, 2001; Oppenheim and Lauder, 2001). Modern electrophysiological studies have revealed that the embryonic motility is produced by spontaneous activity in cranial and spinal motoneurons, which is driven by neural networks in the brainstem and spinal cord (Fortin et al., 1995; O'Donovan, 1999; Marder and Rehm, 2005). One hypothesis of old was that this spontaneous activity is a prototype of the rhythmic discharges observed in the adult central nervous system, such as respiratory and locomotor patterns. Recent advances in anatomical, physiological, pharmacological, and molecular studies, however, have provided another concept: that the embryonic spontaneous activity is a specific phenomenon observed during a restricted period of development, rather than a primordial version of mature central pattern generators.

In this review, we describe characteristics of the spontaneous activity detected from the embryonic brainstem and spinal cord by emphasizing the properties on which the above conclusion is based. In previous studies, spontaneous activity was often analyzed using isolated brainstems or spinal cords, which has caused some confusion, as if the activities in these structures are different, independent phenomena. During early development, in truth, synchronized activity propagates over a wide area of the central nervous system including the brainstem and spinal cord, and thus these regions are functionally correlated. In this review, we summarize the properties of this synchronized activity by focusing on chick, rat, and mouse embryos, in which studies have been most extensively performed. In the following sections, we first provide an overview of the characteristics of the activity in each embryo, and then extract the features common to all three species. This will be followed by a discussion concerning the possible mechanisms underlying the global features. Several excellent reviews related to the present topic have appeared previously (Feller, 1999; O'Donovan, 1999; Ben-Ari, 2001; Chatonnet et al., 2002; Moody and Bosma, 2005; Greer et al., 2006; Ben-Ari et al., 2007; O'Donovan et al., 2008; Blankenship and Feller, 2010), which would help to obtain a better understanding of the issues that are not or only briefly mentioned in this article.

## Synchronized activity in the chick brainstem and spinal cord

### Basic properties and development

The earliest studies of spontaneous activity in the chick embryo involve descriptions of embryonic motility, observed from Hamburger–Hamilton stage 21 (Hamburger and Hamilton, 1951) (3.5 days of incubation: E3.5) as slight flexions of the neck to the left and right, and later two or more S-waves extending from the head to the tail (Hamburger and Balaban, 1963). Electromyography has shown that the activity consists of periodically recurring sequences of activity bursts, which are separated by longer periods of quiescence (Bekoff et al., 1975). Electrophysiological recording of spinal neuronal activity *in ovo* has demonstrated remarkable parallelism between the electrical discharges and embryonic motility, providing direct evidence for the neurogenic basis of the behavior (Ripley and Provine, 1972; Provine, 1973).

**Figure 1:**
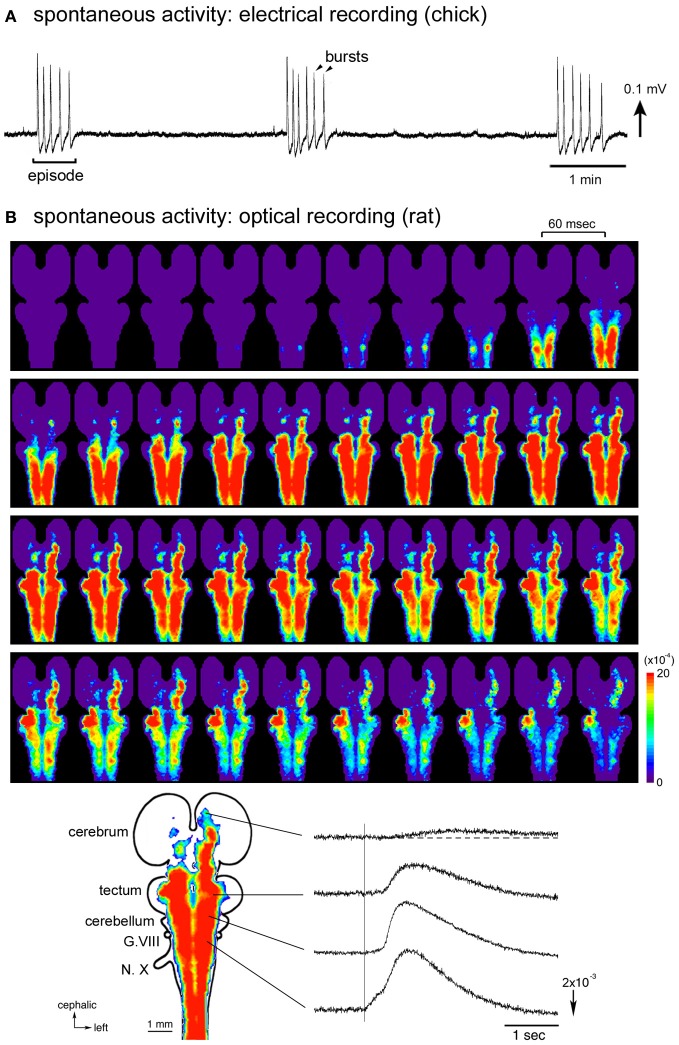
**(A)** Electrical recording of the spontaneous activity in the chick embryo. The signal was recorded from a brainstem-whole spinal cord preparation dissected from a stage 27 (E5.5) embryo with a glass micro-suction electrode applied to the root of the vagus nerve. **(B)** Optical imaging of the spontaneous activity in the rat embryo. The whole brain-spinal cord preparation dissected from an E16 embryo was stained with a voltage-sensitive dye NK2761, and optical recording was made by a 1020-element photodiode array system (Momose-Sato et al., 2001a). At the bottom, a color-coded representation of the maximum signal amplitudes (left) and waveforms (right) of the optical signals are shown. The vertical line in the signal waveforms indicates the onset of the spontaneous electrical discharge detected from the vagus nerve (N.X). Reproduced from Momose-Sato et al. (2007, 2009).

When the brainstem and/or spinal cord is isolated *in vitro*, electrical activity with a similar rhythmic bursting is recorded from the cranial and/or spinal nerves, although the activity pattern is more regular and less variable than *in ovo* (Landmesser and O'Donovan, 1984; Fortin et al., 1995; O'Donovan et al., 1998) (Figure 1A). The accessibility of *in vitro* preparations has entrained the study of spontaneous activity during the early stages of development, and electrophysiological investigations have revealed basic patterns of the activity and their developmental changes. In hindbrain preparations, spontaneous discharges of cranial motor nerves can be recorded from stage 24 (E4) as recurring episodes composed of a single burst of activity (Fortin et al., 1994; Momose-Sato et al., 2009). As development proceeds, substantial changes occur in the activity pattern, including an increase in the interval between episodes (1 min at stages 24–26 and ~6 min at stages 34–36), the number of burst discharges within episodes (single burst at stages 24–26 and more than 15 bursts at stages 34–36), and a decrease in the inter-burst interval within an episode (~8 s at stages 30–32 and ~4 s at stages 34–36), leading to an overall increase in the fraction of time during which the activities are present (Fortin et al., 1994). In the isolated spinal cord, spontaneous activity is detected from the ventral root of the spinal nerve as early as stages 22.5–24 (E3.5–E4) (Milner and Landmesser, 1999; Hanson and Landmesser, 2004). Developmental changes in the pattern of spinal nerve discharges are to a large extent reminiscent of those observed in hindbrain preparations. At stages 25–26, the episode appears at a rate of 1–2/min and consists of a single burst of activity, whereas at stages 36–40, the maximum rate of activity declines to one every 4–5 min and the episode can last 30–40 s with multiple bursts (O'Donovan and Landmesser, 1987; Milner and Landmesser, 1999).

A remarkable feature of the spontaneous activity in the brainstem and spinal cord is the co-activation of different nerves/regions during the early stages of development. Synchronization is observed between paired branchiomotor and spinal nerves, between different rostrocaudal levels, between the left and right sides, and between the nerves innervating the flexor and extensor muscles (Provine, 1973; Fortin et al., 1994, 1995; Milner and Landmesser, 1999). These observations suggest that the early spontaneous activity is widely correlated within the brainstem and spinal cord, possibly by intersegmental and bilateral interactions between different subsets of neurons (Fortin et al., 1995). The correlation of a large number of neurons/areas has been visually demonstrated in optical studies using voltage-sensitive dyes, in which spontaneous and sensory-evoked waves, termed the depolarization waves, propagate over a wide region of the central nervous system including the spinal cord, brainstem, cerebellum, and part of the forebrain (Momose-Sato et al., 2001b, 2003b, 2009).

In addition to the synchronized activity, asynchronous excitation has been detected in the chick brainstem using calcium imaging (Mochida et al., 2009a). In this study, retrogradely labeled reticulospinal and vestibulo-ocular neurons were monitored from stage 20 and stage 25, respectively. Both types of neuron exhibited asynchronous transients from the earliest stage they were monitored, earlier than the emergence of synchronized activity in each population (stage 25 in the reticulospinal neurons and stage 26 in the vestibulo-ocular neurons). The asynchronous and synchronous activities appear to be independent phenomena produced by different mechanisms since (1) the asynchronous activity is resistant to tetrodotoxin, which completely blocks the synchronous activity, and (2) there is no temporal relationship between the two activities or no indication that one evolves from the other (Mochida et al., 2009a). These observations show that, in the early stages of development, individual neurons are spontaneously active independently of each other, and later, become engaged in more widespread synchronous activity. A similar developmental sequence of spontaneous activity has also been reported in the mouse hindbrain (see section Synchronized activity in the mouse brainstem and spinal cord).

### Origin and propagation

Since the synchronized activity recruits a large number of neurons distributed widely along the rostrocaudal axis, it is of interest to identify which part of the central nervous system functions as a generator of the activity. In the isolated hindbrain, when intersegmental relationships are interrupted by transverse sectioning of the hindbrain, the ability to generate the rhythmic pattern is preserved in each transverse slice (Fortin et al., 1995). In addition, single rhombomeres (segmental structures of the early hindbrain) are all able to generate the spontaneous activity when isolated at E2 (stages 10–11) (Fortin et al., 1999; Borday et al., 2003). These observations suggest that any level of the embryonic hindbrain has a capacity to behave as a rhythm generator, and that the specification of rhythmogeneity would take place at a very early stage (Borday et al., 2003).

Spontaneous activity recorded from the isolated spinal cord is considered to be a network-based phenomenon (O'Donovan, 1999). Experiments using combined electrophysiological and optical imaging techniques have shown that, in E8–E11 spinal cords, spontaneous episodic activity appears first in the ventrolateral region of the cord where motoneurons are located. From there, the activity propagates medially to the contralateral side and dorsally on both sides of the cord (O'Donovan et al., 1994; Wenner and O'Donovan, 2001; Arai et al., 2007). Triggering of the episode seems to be mediated by recurrent connections of motoneurons and R-interneurons, the homologue of mammalian Renshaw cells. A plausible scenario is that motoneuron activity monosynaptically excites R-interneurons, which then activate the rest of the network through their depolarizing synaptic action on other neurons within the network, in addition to projecting back to the motoneurons (O'Donovan et al., 2008). Although this scenario is conceivable in developed embryos, the recurrent circuit is in an immature state before E7 (Wenner and O'Donovan, 1999), and the mechanism by which the wave is triggered in the early embryo has yet to be clarified.

What is the relationship between the activities recorded from the isolated hindbrain and spinal cord? Are there multiple generators that drive the hindbrain and spinal networks independently? An optical imaging study using intact brainstem-whole spinal cord preparations has shown that activity arising in one location spreads throughout the brainstem and spinal cord, suggesting that these structures are neither functionally separated nor independent in mediating the correlated wave (Momose-Sato et al., 2009). The origin of the spontaneous wave is in the upper cervical cord/lower medulla near the obex at stages 24–29 (E4–E6) with some variations between the activities. As development proceeds to stages 30–34 (E7–E8), the region responsible for generating the wave shifts caudally, and the activity is initiated in any part of the spinal cord (Momose-Sato et al., 2009).

Taken together, it seems likely that neurons and/or neuronal networks that can produce the spontaneous activity are widely distributed in the brainstem and spinal cord, but the activity in intact preparations is usually paced by a specific region, which probably has the highest excitability. When the primary pacing area is removed, as is the case for the transected hindbrain, other potential generators would become a new initiator of the spontaneous activity.

In another study using a Ca^2+^-imaging technique, Hughes et al. (2009) reported a different spatiotemporal pattern of spontaneous activity in the chick hindbrain. This activity arises in two regions along the midline of the hindbrain, both of which are situated within the caudal group of serotonergic neurons. From these regions, the activity travels in both rostral and caudal directions along the midline. This activity seems to be a homologue of the midline spontaneous activity reported in the mouse embryo, which is driven by the serotonergic neurons located in the medial pons (see section Synchronized activity in the mouse brainstem and spinal cord). In the chick, the midline activity is preceded by a spontaneous wave that originates in the rostralmost spinal cord and travels in the lateral hindbrain (Hughes et al., 2009), suggesting that it occurs secondarily following the widely spreading synchronized activity.

### Pharmacology

To study the role of neurotransmitter systems in mediating the spontaneous activity, pharmacological experiments have been performed. In the spinal cord preparations, spontaneous activity at early developmental stages (stages 25–28: E4.5–E6) is primarily mediated by nicotinic acetylcholine receptors, specifically those that do not contain the α7-subunit (Milner and Landmesser, 1999). Glycine and γ-aminobutyric acid (GABA) also act as excitatory mediators (Milner and Landmesser, 1999; Hanson and Landmesser, 2004), probably because the Cl^−^ reversal potential (E_Cl_) is more positive than the resting potential due to the high intracellular Cl^−^ concentration (Ben-Ari et al., 2007). At stages 25–28, although glutamate receptors are present, their blockers do not affect the spontaneous activity, suggesting that the glutamatergic system does not play a major role in mediating the activity (Milner and Landmesser, 1999).

The pharmacological characteristics of the neural network responsible for the spontaneous activity change during the subsequent stages. The activity in E10–E11 spinal cords is abolished by glutamatergic antagonists, suggesting that, at these stages, glutamate, not acetylcholine, is the primary mechanism that generates the spontaneous activity (Chub and O'Donovan, 1998). An interesting finding of Chub and O'Donovan (1998) was that, when glutamatergic transmission was blocked, after a period of silence, activity reemerged, mediated by another subset of transmitters, GABA and glycine. This implies that the mechanism underlying the spontaneous activity involves interactions of different circuits, and if the primary input is disturbed, another system can substitute to maintain the activity.

The pharmacological nature of the spontaneous activity has recently been re-investigated in the intact brainstem-whole spinal cord, and it has been shown that the switching of transmitters from acetylcholine to glutamate occurs not only in the spinal cord, but also in the brainstem network (Mochida et al., 2009b). In this experiment, both the generation and the propagation of the activity from the brainstem were primarily mediated by nicotinic acetylcholine receptors at stages 26–27 (E5), and glutamate receptors at stages 33–34 (E8), with the transition occurring gradually during E6–E7. GABA and glycine also contributed to the generation of the spontaneous activity, although the propagation network of the brainstem was independent of glycine in the later developmental stages.

In the chick embryo, the dominant neuronal response to GABA and glycine is depolarizing/excitatory, at least until E8 in the brainstem and E10–E11 in the spinal cord (Chub and O'Donovan, 1998; Momose-Sato et al., 1998; Liu et al., 2006). Nonetheless, some neurons in the reticular formation receive hyperpolarizing inputs from GABAergic neurons, which regulate the bursts of the synchronized spontaneous activity (Fortin et al., 1999). In these neurons, the intracellular Cl^−^ concentration is presumably maintained at a low level, and the rebound from the GABAergic inhibitory postsynaptic potential triggers the bursting activity. The expression of the bursting activity is determined by the odd-numbered rhombomeres (r3 and r5) and their interaction with the adjacent even-numbered rhombomeres (Fortin et al., 1999). Experiments manipulating the rhombomere and rhombomere-specific genes have suggested that some molecular cues expressed in rhombomere segments, such as *Krox20*, regulate the expression of bursting patterns of the correlated spontaneous activity (Chatonnet et al., 2002; Borday et al., 2003; Coutinho et al., 2004).

In addition to chemical synaptic antagonists, synchronized activity in the chick brainstem and spinal cord is inhibited by putative gap junction blockers such as octanol, carbenoxolone, mefloquine, and 18ß-glycyrrhetinic acid (Milner and Landmesser, 1999; Momose-Sato et al., 2003a; Hughes et al., 2009; Mochida et al., 2009b). Although interpretation of the results obtained using these blockers is not forthcoming because of the non-specific effects of the drugs on cell membrane conductance (Deutsch et al., 1995; Rouach et al., 2003; Tovar et al., 2009), the results suggest the possibility that the correlated activity is mediated by the coordination of chemical neurotransmitter systems and gap junctional communication.

## Synchronized activity in the rat brainstem and spinal cord

### Basic properties and development

In rats, spontaneous embryonic movements having a close correlation between the forelimb and hindlimb are detected by E (embryonic day: days of pregnancy) 15–E15.5 after acute cesarean section or isolation of the embryo with the uterus (Narayanan et al., 1971; Suzue, 1992). In the dissected brainstem and spinal cord preparations, spontaneous activity is recorded from E13–E13.5 as coordinated rhythmic discharges of cranial and spinal motoneurons (Greer et al., 1992; Nakayama et al., 1999; Ren and Greer, 2003). The motoneuronal discharge is synchronized between different segments of the brainstem and spinal cord and between the right and left sides of the preparation, and does not involve the multiple phases of neuronal activation characteristic of older fetuses, implying that it is distinct from the mature form of activity, such as the respiratory and locomotor rhythms.

In the spinal cord, the coordinated activity is first detected from the cervical and thoracic segments at E13.5, observed throughout the whole spinal cord at E14.5–E17.5, and is lacking or restricted to the thoracic and lumbar segments at E18.5 (Nakayama et al., 1999; Ren and Greer, 2003). During these stages, the interval of the spontaneous activity increases from 2 min at E14.5 to 3–13 min at E17.5, and the activity's duration is lengthened from 2 s at E14.5 to 12–22 s at E17.5. Beyond E17.5–E18.5, spontaneous activity is not detected from the spinal cord (Nakayama et al., 1999; Ren and Greer, 2003), but can be induced by pharmacological manipulations, most commonly by the application of serotonin and *N*-methyl-_D_-aspartate (NMDA) (for a review see Nishimaru and Kudo, 2000). Serotonin-induced motoneuronal discharges alternate between the left and right sides from E18.5, and also between the L2/L3 and L5 segments from E20.5 (Nishimaru and Kudo, 2000). Such alternating activity is similar to the pattern of locomotion, suggesting that the timing of the decline in widespread synchronized activity correlates with the generation of the locomotor network.

In the brainstem-spinal cord preparation, the primordial spontaneous activity disappears after E18, whereas the activity related to respiratory function emerges from E16–E17 (Di Pasquale et al., 1992; Greer et al., 1992; Onimaru and Homma, 2002), in parallel with the morphological and functional differentiation of the pre-Bötzinger complex (E17~: Pagliardini et al., 2003). This also suggests that the disappearance of the primordial synchronized activity is temporally correlated with the generation of the mature central pattern generator. Burst patterns of respiratory motor nerve discharges change drastically between E19 and E20 (Onimaru and Homma, 2002), suggesting that the respiratory network already established at E17 will probably undergo additional changes comparable with the neonatal respiratory network.

### Origin and propagation

Electrophysiological studies of spinal motor patterns have revealed that the location of neural networks initiating the periodic spontaneous bursts shifts from the rostral to caudal region with development (Nakayama et al., 1999; Ren and Greer, 2003). At E13.5–E14.5, the activity appears first in the thoracic cord followed by the other spinal segments, while from E16.5, the lumbar cord leads the thoracic and cervical segments. When the spinal cord is transected, each segment retains spontaneous activity with its own frequency, with the highest rhythmicity being observed in the thoracic cord at E14.5 and the lumbar cord at E16.5. These results suggest that the ability of neurons to generate the spontaneous activity is distributed throughout the spinal cord, and the region with the highest rhythmicity seems to behave as a primary generator of the activity. At E17.5, isolated cervical and thoracic segments are no longer spontaneously active, and at E18.5, the spinal cord becomes silent. This suggests that the loss of spontaneity also develops in a rostrocaudal direction.

Optical imaging studies performed in E15–E16 rat embryos have provided detailed information concerning the spatiotemporal pattern of the synchronized activity (Momose-Sato et al., 2005, 2007). In whole brain preparations, optical signals, either occurring spontaneously or induced by sensory inputs, spread like a wave over a wide region of the central nervous system, recruiting the spinal cord, brainstem, cerebellum, and part of the forebrain (Figure 1B). Concerning the spontaneous wave, the origin of the activity is observed in the cervical, thoracic, and upper lumbar cords with variations at E15, while that at E16 is predominantly identified in the lumbosacral cord. In the E16 preparations, when the oscillatory event occurs, a complex pattern of wave initiation is observed: during the first burst of an episode, spontaneous optical signals appear in the lumbosacral region and propagate rostrally, while with the second burst, the activity is initiated in the cervical cord and propagates rostrocaudally. Thus, at E16, although the primary generator is located in the caudal cord, the cervical cord also plays an important role in induction of the activity, especially in triggering the oscillatory event.

In addition to the spinal cord, the widely spreading spontaneous wave is also initiated in the rostrolateral medulla, which overlaps with the region of the facial nucleus, and dorsomedial pons, although the incidence is low (Momose-Sato et al., 2007). Removal of the primary pacing area by transection of the spinal cord markedly increases the incidence of the wave originating in these regions. These results suggest that neurons and/or neuronal networks located in the medulla and pons also have the ability to produce the synchronized activity, and they would become a dominant generator of the synchronized activity when the influence of the spinal cord is removed.

### Pharmacology

Pharmacological experiments performed in the rat spinal cord have shown that different transmitter systems are responsible for the spontaneous activity age-dependently (Nishimaru et al., 1996; Nakayama et al., 1999; Ren and Greer, 2003). At earlier stages up to E17.5, spontaneous activity is strongly dependent on nicotinic acetylcholine receptors, while at later stages (E18.5~), it is not affected by cholinergic blockers. Antagonists of non-NMDA, but not NMDA, receptors abolish the activity from E16.5 in the cervical cord and E17.5 in the lumbar cord, indicating that the activity becomes mediated by glutamate at the later stage, and that this transition occurs with a rostrocaudal progression. These results show that the dominant transmitter mediating the spontaneous activity switches from acetylcholine to glutamate in a manner reminiscent of that in the chick embryo.

In addition to acetylcholine and glutamate, the spontaneous activity is dependent on glycine and GABA_A_ receptor functions. Activity up to E17.5 is inhibited by glycinergic and GABAergic blockers, while that after E18.5 is augmented by the same drugs (Ren and Greer, 2003). The change in the effects of these blockers is probably due to the change in the cellular response from depolarizing to hyperpolarizing, which is caused by the shift in the Cl^−^ reversal potential. The transition occurs at the same age that the synchronous left/right activity changes into the alternating pattern between the two sides of the cord (Nishimaru and Kudo, 2000). The application of GABA_A_ and glycine receptor antagonists causes the alternating activity to become synchronous (Cowley and Schmidt, 1995), suggesting that the emergence of the locomotor-like pattern is closely coupled with the maturation of inhibitory networks.

Although the primary mechanism for the generation and propagation of the synchronized activity is by chemical transmission, there seem to be additional mechanisms capable of facilitating the spread of the activity. In E16–E18 brainstem-spinal cords, when synaptic transmission is blocked by changing the bathing medium to a low- or zero-[Ca^2+^]_0_ solution, the rhythmic motor discharges are immediately abolished, but within 20 min, a robust, slowly propagating wave reemerges with interburst intervals of 4–6 min and burst durations of 30–50 s (Ren et al., 2006). This wave is insensitive to chemical synaptic antagonists and is dependent on a persistent sodium current (*I*_Nap_). Gap junction blockers abolish the zero-[Ca^2+^]_0_-induced bursting in the nerve roots, but do not block hypoglossal motoneuron bursting as monitored by whole cell recording. It has been proposed that non-synaptically mediated conductance, potentially by extracellular ionic flux and/or electrotonic interaction, acts in concert with neurochemical transmission and gap junctions to promote the spread of the synchronized activity (Ren et al., 2006).

## Synchronized activity in the mouse brainstem and spinal cord

### Basic properties and development

In mice, when an embryo is removed and maintained with transplacental perfusion, spontaneous body movements are observed from E12.5 (Suzue and Shinoda, 1999). In the isolated hindbrain, the electrical recording of activity from cranial nerve roots is possible from E10.5, the stage at which the nerve fibers can be unambiguously identified at the hindbrain's periphery (Abadie et al., 2000). When monitored by the Ca^2+^-imaging method, activity in cranial motoneurons is detected as early as E9.5 (Gust et al., 2003). During E9.5–E10.5, spontaneous activity, either electrical discharges or Ca^2+^ transients, does not show any correlation between the left and right sides, between different cranial nerves, and among motoneuronal cells (Abadie et al., 2000; Gust et al., 2003). At E11.0–E11.5, this asynchronous and tetrodotoxin-insensitive activity changes into synchronous, tetrodotoxin-sensitive activity, which is highly correlated throughout the hindbrain. During E12.5–E13.5, synchronized burst discharges of cranial nerves occur at intervals of ~2 min, but from E14.5 onward, the frequency increases 20-fold (Abadie et al., 2000). In some preparations at E14–E15, a mixed pattern composed of low-frequency bursts and high-frequency discharges is observed (Thoby-Brisson et al., 2005). In mice, respiratory centers become functional from E14.5–E15.5 (Thoby-Brisson and Greer, 2008; Thoby-Brisson et al., 2009), and the expression of the high-frequency bursts seems to be related to the onset of fetal respiratory activity.

In the isolated mouse spinal cord, spontaneous activity is detected from spinal motoneurons from E11.5, a stage when many motoneurons are still migrating and extending their peripheral projections (Hanson and Landmesser, 2003). The episode of activity is highly rhythmic with an interepisode interval of ~2 min at E11.5, which increases to ~8 min at E14.5. Hanson and Landmesser (2003) have shown that the activity is composed of two different spontaneous episodes: a major episode that propagates throughout the cord and is synchronized between the left/right sides as well as among different nerves, and a local episode confined to a single nerve. The examination of motoneuronal responses to antidromic activation has suggested that multiple local circuits composed of motoneurons and GABAergic interneurons might together constitute the overall circuitry, and that their synchronous activation produces the major episode of spontaneous activity.

In the spinal cord, developmental changes in the pattern of spontaneous activity have been studied in detail by comparing the activity in the cervical, thoracic, and lumbar segments (Yvert et al., 2004). Until E13.5, the activity exhibits recurring short episodes that are synchronized over the whole spinal cord. At E14.5, in addition to the short episodes, another type of synchronized activity appears, which is characterized by longer-lasting episodes separated by longer intervals. These longer episodes are specifically observed at E14.5 and are not recorded at any other stage. In the thoracic and lumbar cords, the activity becomes sparse and silent by E16.5. At E17.5, numerous erratic short episodes resume at the thoracic and lumbar levels, while no activity is recorded from the cervical cord. These profiles suggest that the pattern of synchronized activity drastically changes at E14.5. In the mouse spinal cord, bath application of serotonin induces spontaneous activity with a synchronous left/right pattern at E12–E14, a complex pattern at E15–E17, and an alternating locomotion pattern from E18, suggesting that the spinal central pattern generator of locomotion is in the process of development from E15 (Branchereau et al., 2000). Interestingly, E14.5–E15.5 is also the stage when the segregated respiratory network appears in the brainstem (see above), and thus this age may be a critical stage at which mature circuits differentiate to substitute for the larger primordial neuronal assemblies.

### Origin and propagation

**Figure 2:**
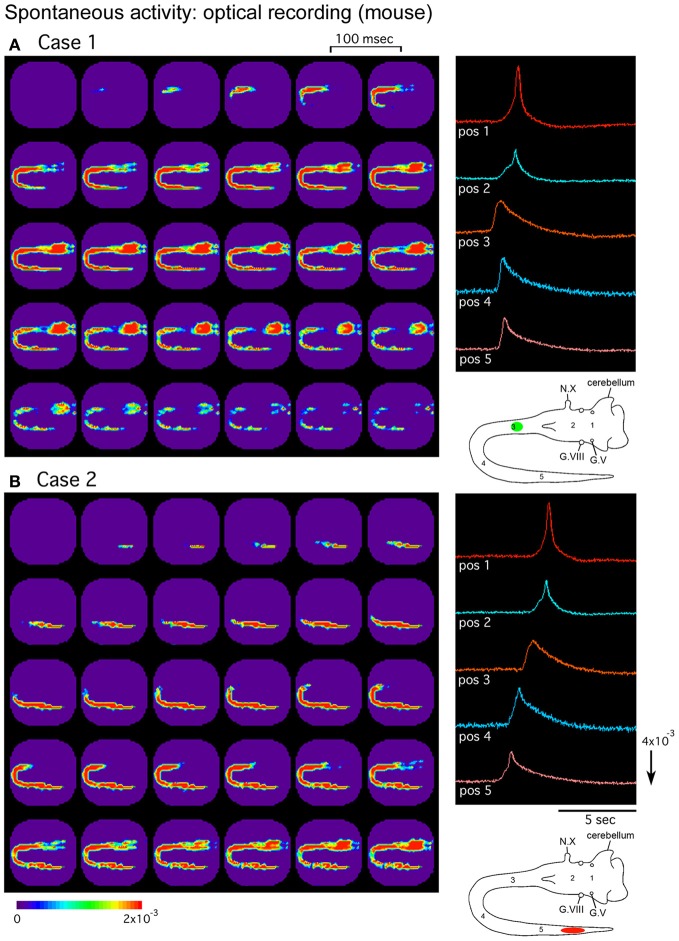
**Pseudocolor images of the spontaneous activity in the mouse embryo.** Spatiotemporal patterns of the spontaneous wave were examined in an E12 brainstem-spinal cord preparation. Images **(A)** and **(B)** were acquired during two independent episodes in the same preparation. The signals on the right were detected from five positions indicated in the lower right insets. In **(A)**, the activity was initiated in the upper cervical cord [green circle in the inset of **(A)**], while in **(B)**, the wave originated in the lumbosacral cord [red oval in the inset of **(B)**]. G.V, trigeminal ganglion; G.VIII, vestibulo-cochlear ganglion; N.X, vagus nerve. Reproduced from Momose-Sato et al. (2012a).

In the mouse embryo, the origins of the spontaneous activity have been studied in different types of preparation. In the isolated hindbrain, Ca^2+^-imaging studies have shown that spontaneous activity originates in the serotonergic neurons located in the midline raphe (Hunt et al., 2005, 2006). In recent experiments using intact brainstem-whole spinal cord preparations, optical imaging with a voltage-sensitive dye has shown that spontaneous activity spreading over the brainstem and spinal cord mainly originates in the cervical and thoracic segments at E11 and lumbosacral cord at E13, with a mixed pattern observed at E12 (Momose-Sato et al., 2012a) (Figure 2). In these preparations, in addition to the widely propagating correlated wave, relatively restricted responses, possibly corresponding to the midline serotonergic activity, have also been detected, suggesting that the synchronized wave and the midline activity reported with Ca^2+^ imaging are distinct phenomena having different spatiotemporal patterns.

**Figure 3:**
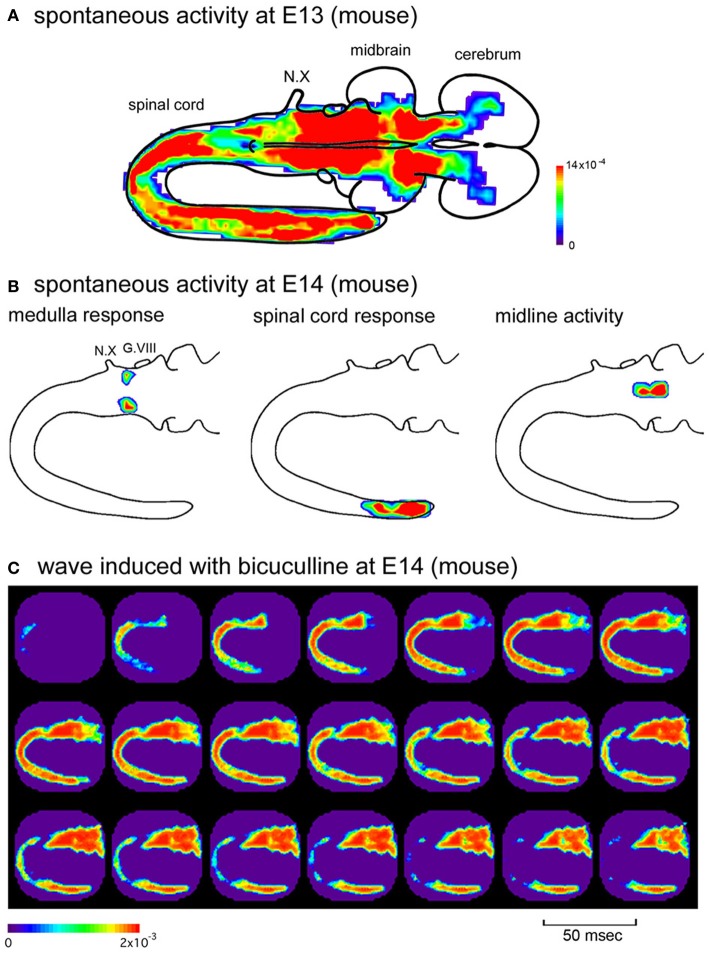
**(A)** Color-coded representation of maximum signal amplitudes of the spontaneous activity in an E13 mouse embryo. **(B)** Spatial distribution of the spontaneous activity in an E14 mouse embryo. The activity was localized to restricted regions of the medulla (left), caudal spinal cord (middle), and midline of the brainstem (right), which corresponds to the parafacial respiratory group, central pattern generator of locomotion, and midline raphe, respectively. The maximum amplitude of the optical signal is presented with color. **(C)** When bicuculline (10 μM) was applied to the E14 mouse preparation, spontaneous activity appeared in the rostral cord and spread over the brainstem and spinal cord, which was similar to the spatiotemporal pattern of the synchronized wave observed at the earlier stages. G.VIII, vestibulo-cochlear ganglion; N.X, vagus nerve. Reproduced from Momose-Sato et al. (2012a,b).

The distribution of the synchronized wave is broad, covering the spinal cord and brainstem at E11, and extending to the forebrain at E12–E13 (Figure 3A) (Momose-Sato et al., 2012a). At E14, the correlation of the brainstem and spinal cord declines, and activity is localized to restricted regions of the medulla and lumbosacral cord (Figure 3B). These profiles show that the widely propagating correlated activity is observed during a specific time window, and the initially synchronized network segregates into more specialized subnetworks at E14.

In another study using Ca^2+^ imaging, spatiotemporal patterns of spontaneous activity were examined in medulla slices, and it was shown that primordial, non-respiratory activity with a low frequency arises in a dorsomedial area and spreads in a ventral direction (Thoby-Brisson et al., 2005). This initiation site is distinct from that of the respiratory-related high-frequency activity observed from E14 onward, which is located in the ventrolateral area corresponding to the differentiating pre-Bötzinger complex. The results show that the neurons in the medulla also have the ability to generate the correlated spontaneous activity when isolated from the spinal cord, similarly to the chick and rat embryos.

In the hindbrain-spinal cord of E12.5–E15.5 mice, Yvert et al. (2011) reported that the movement of artificial cerebrospinal fluid flowing over the ventricle is critical for the expression of the rhythmic waves of activity originating in the hindbrain and propagating in the spinal cord. A similar dependence on fluid movement was also observed for the rhythmic activity recorded in the neonatal mouse cortex (Yvert et al., 2011), suggesting that intracerebral fluid movement may act as a mechanical source underlying the generation of immature rhythmic activity. In the hindbrain-spinal cord preparation, the fluid-dependent activity was induced only when the perfusion inlet was positioned over the pons and medulla, but not on the spinal cord and midbrain (Yvert et al., 2011). Although the relationship of the motion-induced activity with the synchronized wave originating in the spinal cord is not clear, there may be multiple mechanisms generating the activity with similar spatiotemporal patterns.

### Pharmacology

As in the chick and rat, synchronized activity detected from the mouse embryo displays two distinct periods in which different transmitter systems are responsible for generating the activity. In the spinal cord, spontaneous activity is dependent on acetylcholine, glycine, and GABA_A_ receptors during the early phase, E12.5–E14.5 (Hanson and Landmesser, 2003; Myers et al., 2005; Ladle et al., 2007). At these stages, glycine and GABA act as excitatory mediators that promote spontaneous bursts. From E15.5 onward, the acetylcholine-mediated drive is replaced by a glutamatergic drive, and glycine and GABA suppress, rather than promote, the spontaneous activity (Myers et al., 2005; Ladle et al., 2007). This transition seems to occur gradually, spanning at least 1 day of embryonic development. Similar results have been obtained in brainstem-whole spinal cord preparations, although the role of glycine is less obvious, and the switching of the transmitters occurs a little bit earlier, at E12–E13 in the hindbrain and E13–E14 in the spinal cord (Momose-Sato et al., 2012b).

On the basis of the pharmacological characteristics of the spontaneous discharge, a model of the neural circuits that generate the spontaneous activity has been proposed for the E12 spinal cord (Hanson and Landmesser, 2003). In this model, spinal motoneurons, acting via nicotinic acetylcholine receptors containing α7-subunits, play an important role in the generation of the activity. The motoneurons activate other motoneurons and GABAergic interneurons with axon collaterals and provide the excitatory drive needed to elicit a local burst. For these local bursts to propagate throughout the cord, cholinergic transmission dependent on non-α7-subunits, glycinergic depolarization, and possibly gap junctions are required. Given that a local episode is rarely observed spontaneously, it is suggested that the depolarization of the local circuit usually reaches the threshold for propagation throughout the cord.

As discussed above, the widely propagating correlated activity is observed during a specific time window, E11–E13 (E11.5–E13.5). One possible mechanism underlying the substitution of the initially synchronized network with more specialized mature circuits is that the action of GABA changes from excitatory to inhibitory at E14, and this switching is responsible for the decrease in the network excitability of the brainstem and spinal cord, resulting in the disappearance of the large-scale synchronization. It has also been suggested that descending pathways from the brainstem, especially monoaminergic projections, may have some influence (Vinay et al., 2002; Marder and Rehm, 2005). Consistent with the first hypothesis, when GABA_A_ receptor antagonists were applied in the E14 brainstem-whole spinal cord preparation, spontaneous wave appeared in the rostral cord and propagated bidirectionally to the hindbrain and caudal cord (Figure 3C) (Momose-Sato et al., 2012b). These results suggest that the neural network at E14 has the ability to produce the synchronized wave, but is inhibited by GABA. The blockade of GABA receptors disinhibits the neural network, resulting in the generation of the large-scale synchronized wave.

## Global features

In the previous sections, we have described several features of the primordial spontaneous activity detected in the brainstem-spinal cord of chick, rat, and mouse embryos. Widely propagating synchronized activity is generally observed in these embryos, and comparison of the activities between species shows that they share several common characteristics. First, the activity is generated by multiple regions rather than a single, fixed pacemaker. Second, the activity is highly correlated across a large number of neurons and propagates like a wave over a wide region of the central nervous system. Third, the activity is most commonly mediated by multiple neurotransmitters and possibly gap junctions, and the primary mediator switches from acetylcholine to glutamate as development proceeds. Fourth, the activity is expressed during a particular period of development. Finally, the activity is under homeostatic control, which may act to regulate and maintain the overall excitability of the network. Some of these properties have also been described in other developing systems including the retina, cortex, and hippocampus (Wong, 1999; Moody and Bosma, 2005; Torborg and Feller, 2005; Ben-Ari et al., 2007; Allene and Cossart, 2010; Blankenship and Feller, 2010). Spontaneous activity in these structures is beyond the scope of this article and is not reviewed here in detail. Nevertheless, in the following sections discussing the above features, we briefly refer to the comparable properties observed in other systems, in addition to the evidence and suggestions provided for the brainstem and spinal cord.

### Generation of the activity

In general, there are two hypotheses regarding the mechanisms underlying the genesis of the spontaneous activity: one is the pacemaker hypothesis, in which the rhythm arises in pacemaker neurons, and the other is the network hypothesis, in which the activity is generated by network interactions. Spontaneous activity in the developing circuits seems to be generated by various combinations of pacemaker-like intrinsic membrane properties and network interactions (Blankenship and Feller, 2010). In the embryonic brainstem and spinal cord, the origin of the large-scale synchronized activity is not fixed, but varies from event to event and depending on the developmental stage. Moreover, the activity is retained in transected segments at various levels of the brainstem and spinal cord. These properties support the idea that the activity does not rely on a single pacemaker, although the possibility cannot be ruled out that neurons with pacemaker-like properties are widely disseminated in a register along the neuraxis.

The mechanism by which a developing network generates rhythmic activity may depend on hyperexcitability of the early network, which results from the depolarizing nature of the major transmitters including GABA and glycine, and recurrent excitatory connections between neurons (Feller, 1999; O'Donovan, 1999). Concerning the mechanism dictating the periodicity, a model has been proposed by O'Donovan and colleagues based on work on the embryonic chick spinal cord (O'Donovan et al., 1998; Tabak et al., 2001; see also Godfrey and Eglen, 2009; Ford et al., 2012 for the model of retinal waves). In this model, activity-dependent depression occurs after an episode of activity: the membrane is hyperpolarized, and the evoked and spontaneous synaptic potentials fall to a minimum. One mechanism generating the post-episode depression is chloride's extrusion through the GABA_A_ receptor-gated chloride channel (Chub and O'Donovan, 2001; Chub et al., 2006). During the interepisode interval, the intracellular chloride concentration is restored, and network excitability recovers gradually. When the network excitability reaches a threshold, neurons will be explosively recruited throughout the network, and an episode will recur with a time constant of minutes. One important characteristic of this model is that activity can be initiated in any part of the preparation where the excitability is sufficiently high to sustain regenerative recruitment of the entire network, and no exact, highly precise circuit needs to exist to produce the activity pattern (O'Donovan et al., 1998; Feller, 1999). This model of activity-dependent depression also seems to be applicable to the mammalian spinal cord, in which the network undergoes refractoriness following an episode of activity (Hanson and Landmesser, 2003).

The origin of the correlated spontaneous activity is usually located in the rostral cord at the early stage and shifts caudally with development. Possible mechanisms underlying the shift in the origin include a decrease and an increase in neural excitability in the rostral and caudal spinal cords, respectively, assuming that the wave is paced by the region with the highest excitability. In the chick embryo, it has been reported that, in the early developmental stage (E4–E5), electrical stimulation is effective in inducing the evoked wave only when it is applied to the upper cervical cord and lower medulla near the obex, while at E7–E8, regional differences in neural excitability are less prominent (Momose-Sato et al., 2009). This profile suggests that there is a gradient of neural responsiveness along the rostrocaudal axis of the spinal cord, and that this gradient changes dynamically with development.

In recent studies in the hippocampus, it has been reported that early-born neurons play more important roles in the generation of synchronized network activity, and that the temporal origin is a critical determinant of cell function (Picardo et al., 2011; Marissal et al., 2012). Morphologically, cervical motoneurons are produced slightly earlier than lumbar motoneurons in the spinal cord (Nornes and Das, 1974; Hollyday and Hamburger, 1977; Altman and Bayer, 1984). However, it is known that some cranial motoneurons and neurons in the reticular formation differentiate earlier than the spinal motoneurons (McConnell and Sechrist, 1980; Altman and Bayer, 1984). Considering these findings, it seems likely that the origin of the correlated activity is not simply determined by the chronological sequence of morphogenesis, but requires other factors possibly involving the maturation of intrinsic excitability and intercellular communication systems.

### Large-scale propagation

Optical imaging using Ca^2+^ indicators and voltage-sensitive dyes has revealed that the activity spreads like a wave over a wide region of the central nervous system, maximally extending to the lumbosacral cord and to the forebrain, suggesting the possibility that the activity not only regulates the development of the brainstem and spinal cord, but also affects some developmental processes in the higher brain. The propagation velocity of the widely spreading spontaneous wave is 4–22 mm/s measured in the brainstem and spinal cord of chick and rat embryos (O'Donovan et al., 1994; Arai et al., 2007; Momose-Sato et al., 2007). This propagation is too slow to be explained by axonal conduction along the unmyelinated fiber at the corresponding stage (0.1–0.2 m/s: Sakai et al., 1991). Potential mechanisms underlying the spread of the activity include sequential synaptic activation of adjacent regions coupled by short-range synaptic connections (Fortin et al., 1995; O'Donovan et al., 2008), and the coordination of chemical transmitters with gap junctions as well as electrical interactions between neighboring neurons (Hanson and Landmesser, 2003; Ren et al., 2006).

In addition to mediating fast synaptic transmission, neurotransmitters act as biomedical signals during development (Lauder, 1993; Nguyen et al., 2001; Luján et al., 2005). Non-synaptic transmission has been suggested to play an important role in propagation of the depolarizing wave (Demarque et al., 2002). In the mouse spinal cord, it has been demonstrated that a non-synaptic release of glycine influences the excitability of spinal neurons, thereby modulating the propagation of the spontaneous activity (Scain et al., 2010). Glycine is released from radial cells by a non-vesicular mechanism, depending on volume-sensitive chloride channels. Since glycine is important for the local burst to spread throughout the cord in the mouse (Hanson and Landmesser, 2003), the non-synaptic release of transmitters may be one of the mechanisms underlying the large-scale propagation of the primordial activity.

In the developing neocortex and hippocampus, sequential expression of uncorrelated and correlated activity has been described (Crépel et al., 2007; Allène et al., 2008; Allene and Cossart, 2010). Initially expressed spontaneous activity usually consists of sporadic calcium spikes that are poorly correlated between neurons. This is followed by coherent, gap junction-dependent non-synaptic activity, which involves small groups of neurons producing calcium plateaus and is termed synchronous plateau assemblies (SPAs). With further development, synapse-dependent synchronized activity emerges, which recruits a large number of neurons and is referred to as giant depolarizing potentials (GDPs), cortical early network oscillations (cENOs), and others. Early expression of the asynchronized activity prior to the correlated wave observed in the chick and mouse brainstem seems to be consistent with the developmental sequence identified in the neocortex and hippocampus. In the hippocampus, it has been reported that the SPAs and GDPs are mutually exclusive, and that disappearance of the SPAs is required for the expression of the GDPs (Crépel et al., 2007). In the brainstem and spinal cord, activity corresponding to the SPAs has not been clearly identified because the correlated activity is sensitive to both chemical transmitter antagonists and gap junction blockers from the earliest stage of expression. It is thus difficult to determine whether a similar mechanism to that in the higher brain is operating in the brainstem and spinal cord.

### Switching of the pharmacological nature

The widely spreading synchronized activity in the brainstem and spinal cord is mediated by multiple neurotransmitters, and its pharmacological nature undergoes developmental changes. During the early phase, nicotinic acetylcholine receptors play a significant role in mediating the activity, while at the later stage, the activity is dominated by glutamatergic transmission. The second type of switching occurs in the GABAergic and glycinergic systems, in which the neural response changes from depolarization/excitation to hyperpolarization/inhibition.

The switching of major transmitters mediating the correlated activity from acetylcholine to glutamate has been well documented for the retinal wave (Catsicas et al., 1998; Wong et al., 1998; Wong, 1999; Zhou and Zhao, 2000; Torborg and Feller, 2005). In the higher brain, nicotinic acetylcholine receptors do not seem to play a significant role, and the synapse-driven network activity is dependent on either GABA_A_ or glutamate receptors. In the hippocampus, GABAergic synapses are established before glutamatergic ones, and the first synapse network-dependent spontaneous activity, the GDPs, is mediated by GABA_A_ receptors, which are later replaced with glutamatergic activity (Garaschuk et al., 1998). A similar developmental sequence has been reported in the mouse cerebrum (Conhaim et al., 2011), but not in the rat neocortex, in which the cENOs driven by glutamatergic transmission precede the GABAergic activity referred to as the cortical GDPs (cGDPs) (Allène et al., 2008). In the brainstem and spinal cord, GABA, together with acetylcholine, plays a significant role from the earliest stage of wave expression, suggesting that the GABAergic network is established prior to the glutamatergic system, as in the hippocampus.

In the process of functional synaptic formation in the brainstem and spinal cord, several studies have reported earlier expression of GABAergic signaling than glycinergic synaptic currents (Kotak et al., 1998; Gao et al., 2001; Awatramani et al., 2005). Even in a situation in which the glycinergic synapses have not been differentiated, glycine contributes to the correlated activity by a non-synaptic mechanism (Scain et al., 2010). GABA and glycine are both involved in the correlated activity in the brainstem and spinal cord, but their relative contributions have varied between investigations, so it is difficult to draw a definitive conclusion concerning which is the major transmitter at each stage.

With regard to the mechanisms by which the pharmacological characteristics of the spontaneous activity change during development, interactions of different transmitter systems have been suggested. Using choline acetyltransferase (ChAT)-deficient mouse spinal cord, Myers et al. (2005) have found that cholinergic signaling determines the timing of glutamatergic activity and also the transition of glycinergic responses from excitation to inhibition. Expression of the glutamatergic drive appears to precede the switching of glycine function, and thus it is speculated that the cholinergic activity dictates the glutamatergic function, which, in turn, leads to the maturation of inhibitory signaling. In another study using the chick embryo *in ovo*, Liu et al. (2006) have shown that the chronic blockade of nicotinic acetylcholine receptors prevents the conversion of GABA/glycinergic responses from excitation to inhibition. In the spinal cord, the change in pharmacological nature occurs around the time when motoneuron axons reach their muscle targets, and it is possible that target-derived signals, such as brain-derived neurotrophic factor (BDNF), act as another factor regulating the switching of the transmitter (Fiumelli and Woodin, 2007).

### Critical period of activity

Primordial correlated activity is observed during a particular period of development. In the spinal cord, the early coordinated network segregates into more specialized domains with development, and the synchronized activity is later replaced by alternating locomotor-like activity. In the rodent brainstem, the primordial activity disappears around the stage at which the respiratory network is differentiated in the pre-Bötzinger complex and parafacial respiratory group (Borday et al., 2003; Pagliardini et al., 2003; Thoby-Brisson et al., 2005; Greer et al., 2006).

As possible mechanisms underlying these changes, several factors have been suggested to play a key role, including a shift in GABA/glycine responses from depolarization to hyperpolarization, and an influence of neuromodulators such as serotonin (Whelan, 2003; Marder and Rehm, 2005; Momose-Sato et al., 2012b). The link between the establishment of the mature centers and the preexisting immature activity is currently unknown. According to the findings obtained in the mouse embryo, the respiratory generator does not seem to be simply derived from the cells generating the immature activity, since these two populations can coexist in the same preparation with non-overlapping territories (Thoby-Brisson et al., 2005). The development of the primordial activity and that of the respiratory function are both dependent on the rhombomere and segmentation genes such as *Krox20* and *Hoxa2* (Chatonnet et al., 2002; Borday et al., 2006), and it would be interesting to identify whether the transition from the primordial to mature form of activity is under the influence of genetic control.

Developing neurons have electrical properties different from mature neurons (Moody and Bosma, 2005). Changes in the properties and distribution of voltage-, Ca^2+^-, and ligand-gated channels as well as gap junctions may result in the restricted and selected recruitment of neuronal circuitry and then minimize the spatial spread of spontaneous activity. The transition from an immature to mature state of channel expression is activity-dependent (Moody and Bosma, 2005), and thus it is possible that termination of the primordial activity is self-regulated by its own activity patterns.

### Homeostatic control

There is growing evidence that immature network activity is homeostatically regulated (Davis and Bezprozvanny, 2001; Corner et al., 2002; Turrigiano and Nelson, 2004; Maffei and Fontanini, 2009; Blankenship and Feller, 2010). The removal of a crucial component of a circuit presenting spontaneous activity often tends to be compensated for by the remaining components. With regard to the brainstem-spinal cord network, one of the earliest indications of such compensation was provided by Chub and O'Donovan (1998), who showed that the pharmacological blockade of glutamatergic transmission in E10–E11 chick spinal cords results in a cessation of the spontaneous activity, but after several hours, the activity is reestablished through GABAergic and glycinergic systems. A similar phenomenon has also been observed in E3–E4 chick spinal cords: when glutamate, GABA, and nicotinic acetylcholine receptors are blocked, the spontaneous activity is transiently eliminated, but resumes through muscarinic acetylcholine receptor function (Milner and Landmesser, 1999). When neural transmission is chronically inhibited by the *in ovo* application of blockers, similar compensatory regulation is observed (Gonzalez-Islas and Wenner, 2006; Wilhelm et al., 2009). Possible mechanisms underlying such homeostatic regulation involve increases in cellular excitability by changing sodium and potassium currents and changes in synaptic strength by synaptic scaling (Gonzalez-Islas and Wenner, 2006; Wilhelm et al., 2009). As discussed above, the *in ovo* application of nicotinic acetylcholine receptor antagonists prevents the switching of GABA/glycine responses from excitation to inhibition (Liu et al., 2006), and genetic manipulation of choline acetyltransferase causes the precocious expression of glutamatergic activity (Myers et al., 2005). These changes might also be another form of compensatory response advantageous for maintaining the level of network activity.

Compensation of the network activity is not only observed between the transmitter systems but also occurs in non-synaptic networks. In the embryonic rat brainstem-spinal cord, the blockade of synaptic transmission by a zero-[Ca^2+^]_0_ solution abolishes the spontaneous activity, but is followed by the emergence of a new form of activity mediated by non-synaptic mechanisms, possibly including gap junctions and electrotonic interactions between neighboring neurons (Ren et al., 2006). It thus seems likely that interactions and the coordination of multiple elements of the network are determinants of the homeostatic regulation.

In addition to the compensatory regulation for the pharmacological blockade, spontaneous activity is homeostatically maintained following deprivation of the primary rhythm generator. When the function of the primary pacing area or pathway propagating from there is disturbed, other regions become a new generator and produce the activity with a similar pattern to the previous one (Momose-Sato et al., 2007). This can be achieved because most regions of the brainstem and spinal cord have the ability to produce the spontaneous activity, and the network is likely to behave as a self-distributing system.

The homeostatic regulation could serve two competing functions: maintaining an adequate level of excitation and limiting the amount of synchrony to prevent epileptic overexcitation. These two requirements might be balanced to sustain and stabilize the network activity in the face of environmental perturbation.

## Functional significance and future perspectives

Recent advances in our understanding of the widely propagating synchronized activity in the brainstem and spinal cord are due to progress using multiple techniques including electrophysiology, molecular biology, and optical imaging. Although much consensus has been obtained concerning the global features of the activity, there are still several unanswered questions that should be addressed in future investigations.

Perhaps the most important issue is the functional significance of the activity. Behaviorally, the spontaneous correlated activity in the brainstem and spinal cord is associated with body movement observed *in ovo* and *in utero*, and is likely to regulate muscle contractions by outputs conducted through the cranial and spinal motor nerves. Muscle paralysis by chronic blockade of the activity by the *in ovo* application of drugs results in the deficiency of muscle and bone development (Roufa and Martonosi, 1981; Persson, 1983; Hall and Herring, 1990). In experiments using transgenic mice that lack glutamate decarboxylase (GAD), it has been shown that inhibition of the hindbrain correlated activity results in impairment of mouth and tongue movements, causing facial malformation involving a cleft palate (Tsunekawa et al., 2005). Such behavioral control of embryonic phenotypes might be one role of the correlated brainstem and spinal activity, although other roles should be considered because the activity includes a large population of neurons other than motoneurons. At the later developmental stage, it has been suggested that fetal/neonatal motility has some similarities with movement related to active sleep (Corner, 1977). However, the relationships of primordial embryonic activity with sleeping behavior have yet to be clarified.

In addition to generating somatic commands, the brainstem functions as an autonomic center that controls internal environments and body conditions. Spontaneous activity in the brainstem may facilitate early differentiation of the neural networks related to the autonomic function, which seems to be indispensable for the survival and growth of the embryo.

Generally, the spontaneous activity in the developing nervous system is considered to play a fundamental role in activity-dependent processes of neural circuit formation (Feller, 1999; Moody and Bosma, 2005; Blankenship and Feller, 2010). In the embryonic spinal cord, several investigations have provided evidence that the primordial spontaneous activity is indispensable for proper development of the neural network and expression of behavior. For example, in the chick spinal cord, blocking or slowing the spontaneous activity during stages 20–30 (E3–E7) causes dorsal-ventral pathfinding errors of motoneuron axons together with down-regulation of adhesion/guidance molecules such as polysialic acid and EphA4, suggesting that the formation of spinal motor circuits is precisely regulated by the frequency and pattern of spontaneous activity (Hanson and Landmesser, 2004; Hanson et al., 2008; Kastanenka and Landmesser, 2010). Application of the drugs at more developed stages (E8–E10) results in compensatory changes in motoneuron excitability and synaptic strength, suggesting that spontaneous activity plays an important role in the coordinated maturation of synapses on motoneurons (Gonzalez-Islas and Wenner, 2006; Wilhelm et al., 2009). In choline acetyltransferase (ChAT)-mutant mice, elimination of acetylcholine-mediated activity results in a deficiency in the development of locomotor function (Myers et al., 2005). Although spontaneous activity in the amphibian spinal cord is not the subject of this review, a series of elegant experiments in the Xenopus spinal cord and related studies should be mentioned when considering the role of primordial activity in developmental processes including the specification of transmitter phenotypes (Spitzer et al., 2004; Demarque and Spitzer, 2012).

Despite these demonstrations, the functional significance of the activity, especially the necessity of its large-scale correlation over a wide region of the central nervous system, is still unresolved. Primordial activity having characteristics similar to those described here has been observed in several other structures of the developing nervous system (Wong, 1999; Moody and Bosma, 2005; Torborg and Feller, 2005; Ben-Ari et al., 2007; Allene and Cossart, 2010; Blankenship and Feller, 2010), and investigations from multiple perspectives would improve our understanding of the activity and its role in the development of the nervous system.

### Conflict of interest statement

The authors declare that the research was conducted in the absence of any commercial or financial relationships that could be construed as a potential conflict of interest.

## Supplementary material

Movie S1 **Embryonic motility of an E6 chick embryo observed *in ovo***.

## Abbreviations

cENOs: cortical early network oscillations
E: embryonic day, which shows days of incubation in chicks and days of pregnancy in rats and mice
GABA: γ-aminobutyric acid
GDPs: giant depolarizing potentials
NMDA: *N*-methyl-_D_-aspartate
SPAs: synchronous plateau assemblies.
